# Interrogation of Carboxy-Terminus Localized *GJA1* Variants Associated with Erythrokeratodermia Variabilis et Progressiva

**DOI:** 10.3390/ijms23010486

**Published:** 2022-01-01

**Authors:** Sergiu A. Lucaciu, Qing Shao, Rhett Figliuzzi, Kevin Barr, Donglin Bai, Dale W. Laird

**Affiliations:** 1 Department of Anatomy and Cell Biology, University of Western Ontario, London, ON N6A 5C1, Canada; slucaciu@uwo.ca (S.A.L.); cindy.shao@schulich.uwo.ca (Q.S.); rfigliu2@uwo.ca (R.F.); kevin.barr@schulich.uwo.ca (K.B.); 2 Department of Physiology and Pharmacology, University of Western Ontario, London, ON N6A 5C1, Canada; donglin.bai@schulich.uwo.ca

**Keywords:** connexin43 (Cx43), epidermis, keratinocyte, erythrokeratodermia variabilis et progressiva (EKVP), gap junctional intercellular communication (GJIC), internalization, degradation, variants

## Abstract

Although inherited *GJA1* (encoding Cx43) gene mutations most often lead to oculodentodigital dysplasia and related disorders, four variants have been linked to erythrokeratodermia variabilis et progressiva (EKVP), a skin disorder characterized by erythematous and hyperkeratotic lesions. While two autosomal-dominant EKVP-linked *GJA1* mutations have been shown to lead to augmented hemichannels, the consequence(s) of keratinocytes harboring a de novo P283L variant alone or in combination with a de novo T290N variant remain unknown. Interestingly, these variants reside within or adjacent to a carboxy terminus polypeptide motif that has been shown to be important in regulating the internalization and degradation of Cx43. Cx43-rich rat epidermal keratinocytes (REKs) or Cx43-ablated REKs engineered to express fluorescent protein-tagged P283L and/or T290N variants formed prototypical gap junctions at cell–cell interfaces similar to wildtype Cx43. Dye coupling and dye uptake studies further revealed that each variant or a combination of both variants formed functional gap junction channels, with no evidence of augmented hemichannel function or induction of cell death. Tracking the fate of EKVP-associated variants in the presence of the protein secretion blocker brefeldin A, or an inhibitor of protein synthesis cycloheximide, revealed that P283L or the combination of P283L and T290N variants either significantly extended Cx43 residency on the cell surface of keratinocytes or delayed its degradation. However, caution is needed in concluding that this modest change in the Cx43 life cycle is sufficient to cause EKVP, or whether an additional underlying mechanism or another unidentified gene mutation is contributing to the pathogenesis found in patients. This question will be resolved if further patients are identified where whole exome sequencing reveals a Cx43 P283L variant alone or, in combination with a T290N variant, co-segregates with EKVP across several family generations.

## 1. Introduction

Connexins (Cx) are a family of hemichannel and intercellular gap junction channel-forming proteins that enable cells to communicate with the extracellular milieu and adjoined cells, respectively. Gap junction channel transfer of ions and small molecules of up to 1 kDa, termed gap junctional intercellular communication (GJIC), occurs in virtually all cell types found in the body, including keratinocytes, and is required for normal tissue physiology and homeostasis [1]. Consequently, most cells express multiple members of the connexin family that often intermix to form complex channel configurations with varying permeability and gating properties [1,2].

Interestingly, as keratinocytes of the human epidermis progress through a terminal differentiation program called keratinization, they modulate the expression of at least eight connexin isoforms, namely Cx26, Cx30, Cx30.3, Cx31, Cx32, Cx40, Cx43, and Cx45 [3,4,5,6]. In this context, we refer to them as ‘keratinocyte connexins’. Mutations in the genes that encode five of these keratinocyte connexins are clinically linked to multiple cutaneous disorders with varying degrees of severity [7]. One of these disorders is erythrokeratodermia variabilis et progressiva (EKVP), a rare hereditary skin condition characterized by distinct and overlapping erythema and hyperkeratosis that are usually present at birth or develop during infancy, and worsen with age [8]. While these lesions are not life-threatening, they cause substantial morbidity due to pain and irritation. EKVP is genetically heterogenous with linkages to seven genes that include three connexin-encoding genes: *GJB3* (Cx31) [9,10,11,12,13], *GJB4* (Cx30.3) [4,14,15,16], and *GJA1* (Cx43) [17,18].

There are ~100 mutations/variants in *GJA1* identified to date that are associated with at least six disorders [19]. The vast majority of *GJA1* mutations cause oculodentodigital dysplasia (ODDD), a disorder that commonly presents with craniofacial abnormalities, digit fusion, and developmental defects of the eyes and teeth [20]. On rare occasions, ODDD presents with a skin phenotype in the form of palmoplantar keratoderma (PPK) [21,22]. In 2015, the first two *GJA1* mutations (p.A44V and p.E227D) were linked to EKVP [17]. Affected individuals with either mutation had normal skin at birth that progressed to PPK, hyperkeratosis, and transient figurate erythema at sites of friction [17]. When expressed in HeLa cells, the Cx43 mutants assembled into prototypical gap junction plaques with normal gap junction channel properties, but also formed augmented hemichannels when expressed in *Xenopus* oocytes [23]. These findings align well with hyperactive or leaky hemichannels underpinning a number of connexin-based inherited skin pathologies [19,23,24,25].

Recently, two novel de novo autosomal dominant missense variants in *GJA1* (p.P283L and p.T290N) were identified in two unrelated Chinese patients with EKVP [18]. In both cases, these variants were not detected in unaffected family members and 100 unrelated controls, and no variants were identified in *GJB3* or *GJB4* [18], although other genes linked to EKVP were not investigated [26,27,28,29,30]. Notably, this study reported the first case of EKVP associated with a *GJA1* double variant (p.P283L and p.T290N) being found in the same patient, though it is unclear if both variants occurred on the same or different alleles [18]. The patient harboring the P283L variant developed erythema on the hands and feet [18]. In contrast, the patient with both P283L and T290N variants had more severe disease, with widespread erythematous and hyperkeratotic lesions on the face, neck, limbs and inguinal region with progressive PPK [18]. Low resolution immunofluorescent analysis of skin biopsies suggested that these variants, along with endogenous Cx43 encoded by the unaffected allele, may be intracellularly retained [18], although this was not entirely clear.

Structurally, the p.P283L and p.T290N variants occur within a carboxy terminus motif of Cx43 that is the binding site for several members of the Cx43 interactome [31]. Notably, the p.P283L variant occurs within the binding sequences for c-Src, phosphatase and tensin homolog (PTEN), C-terminal c-Src kinase (Csk), and the E3 ubiquitin ligase NEDD4 [31,32,33,34,35,36]. A neighboring Y286A substitution increased the half-life of Cx43 from 2 h to 6 h, identifying the tyrosine-based sorting signal as important for the clearing of Cx43 from the cell surface [36]. Prior to being identified as an EKVP-associated variant, the P283L amino acid substitution was shown to cause a modest increase in the steady-state levels when expressed in SKHep1 cells compared to wild-type Cx43 [36]. Due to the steric properties of proline residues, the P283L amino acid substitution may have altered the accessibility and functionality of proximal Cx43 binding sequences. Interestingly, the P283 site occurs in close proximity to the binding sites of Hsc70 and the µ2 subunit of the AP-2 adapter complex, which have been reported to be involved in the turnover of Cx43 [36,37,38]. Two MAPK phosphorylation sites that regulate NEDD4 binding are also found upstream at residues S279 and S282 [31,32,36,39]. Thus, we speculated that the EKVP patient that harbors both the P283L/T290N variants could have additive effects possibly explaining the apparently more severe phenotype [18].

In the current study, we examined the cellular distribution, trafficking, stability, and functional status of the P283L and T290N variants individually and in combination. To avoid a reliance on connexin-deficient reference cells from tissue unrelated to the skin, we employed spontaneously immortalized rat epidermal keratinocytes (REKs) that retain the capacity to fully differentiate into organotypic epidermis [40,41]. In some cases, REKs were used that were CRISPR/Cas9 engineered to ablate Cx43 expression, to allow for the functional status of the variants to be assessed in keratinocytes in the absence of endogenous Cx43 [40].

## 2. Methods

### 2.1. Cell Culture, DNA Constructs, and Transfections

Wildtype rat epidermal keratinocytes (REKs), Cx43-ablated REKs, and HeLa cells were grown in Dulbecco’s Modified Eagle’s Medium (DMEM, ThermoFisher, Burlington, ON, Canada, Cat# 11960) supplemented with 2 mM glutamine, 10% fetal bovine serum, and 100 U/mL penicillin and streptomycin. Cells were cultured at 37 °C and 5% CO_2_ and subcultured once they reached 70–80% confluency.

The generation of plasmids encoding P283L, T290N, and P283L/T290N (both variants engineered on the same plasmid) within human Cx43 tagged to moxGFP was outsourced to NorClone Biotech Labs (London, ON, Canada) for site-directed engineering and sequence confirmation. For transfections, cultured HeLa cells, REKs, or REKs with endogenously knocked-out Cx43 (Cx43–KO REKs) were transiently transfected with 1 µg DNA using Lipofectamine 3000 (ThermoFisher, Cat# L3000015) as per the manufacturer’s instructions. To further model the compound heterozygous state, given the ambiguity as to whether both variants were found on the same or different alleles, cells were also transiently co-transfected as described above, with 0.5 μg of each plasmid encoding P283L or T290N (denoted as P283L + T290N).

### 2.2. Immunolabeling and Microscopy

Transfected REKs or Cx43-ablated REKs plated on glass coverslips were fixed in ice cold 80% methanol/20% acetone solution for 15 min at 4 °C and subsequently blocked with 2% bovine serum albumin (BSA; MilliporeSigma, Oakville, ON, Canada, Cat# A2153) for 30 min at room temperature. Subsequently, cells were washed and incubated for one hour at room temperature in primary antibody solutions consisting of either mouse monoclonal anti-protein disulfide isomerase (PDI; Enzo Life Science, Burlington, ON, Canada, Cat# ADI-SPA-891F), mouse monoclonal anti-GM130 (BD Transduction Laboratories, San Jose, CA, USA, Cat# 610822) (both diluted 1:200), or rabbit polyclonal anti-Rab7 (Santa Cruz Biotechnology, Dallas, TX, USA, Cat# 376362) (1:100) to demarcate the ER, Golgi apparatus, and endosomes, respectively. In some cases, cells were incubated, as described above, with rabbit polyclonal anti-Cx43 (MilliporeSigma, Cat# C6219) (1:200) to stain for endogenous Cx43. Cells were subsequently washed and incubated in Alexa Fluor 555-conjugated goat anti-mouse IgG (ThermoFisher, Cat# A32727) (1:800) or Alexa Fluor 555-conjugated goat anti-rabbit IgG (ThermoFisher, Cat# A32732) (1:800) secondary antibodies for 1 h. All coverslips were washed and counterstained with Hoechst 33342 (ThermoFisher, Cat# H3570) (1:1000) to demarcate the nuclei and mounted onto microscope slides using Airvol mounting medium. Confocal images were captured on a Zeiss LSM800 confocal microscope.

To assess the ability of each variant to assemble into gap junctions, a third-party investigator that was blinded to the treatment was used to quantitate the number of gap junction plaques at cellular interfaces of apposed transfected cells. A gap junction plaque was defined as a linear punctate green fluorescence signal of greater than 0.2 μm situated at a cell–cell interface between two transfected cells. A total of five independent transfections were performed, where approximately 10–30 cell pairs were quantified.

### 2.3. Fluorescence Recovery after Photobleaching

Cx43-ablated REKs were seeded into 35-mm glass bottom dishes (VWR, Mississauga, ON, Canada, Cat# 10810-056) and incubated for 24 h at 37 °C and 5% CO_2_. Each dish was loaded with CellTrace™ Calcein Red-Orange, AM (ThermoFisher, Cat# C34851) (5 μg/mL) for 10 min at 37 °C and 5% CO_2_. REKs were rinsed three times in PBS before adding room temperature Opti-MEM (ThermoFisher, Cat# 31985070) and transferring to a live cell imaging apparatus maintained at 37 °C. Once enzymatically modified, calcein red-orange is trapped inside live cells and can only pass to an adjoining cell via functional gap junction channels. Randomly selected single cells within small cell clusters were imaged prior to bleaching on a Zeiss LSM 800 confocal Airyscan microscope equipped with a 63× oil immersion objective lens. A 561-nm argon laser was used to photobleached cells to ~20% of their initial fluorescence intensity. Fluorescence recovery, due to the passage of calcein red-orange from contacting cells, was imaged at 1 s intervals for 2 min and quantified using the Time Series Analyzer v3 (https://imagej.nih.gov/ij/plugins/time-series.html, accessed on 22 September 2021) plugin for ImageJ (U. S. National Institutes of Health, Bethesda, MD, USA, https://imagej.nih.gov/ij/, accessed on 22 September 2021). Fluorescence recovery as defined by the area under the curve (AUC) was calculated and normalized to the fluorescence recovery of wildtype Cx43 at 120 s. Data were analyzed using a one-way ANOVA with Tukey’s post-hoc test.

### 2.4. Scrape Load Dye Transfer Assay

Control, Cx43-ablated REKs, and Cx43-ablated REKs engineered to express wildtype Cx43 or Cx43 variants were grown to confluency in cell culture treated six-well plates (ThermoFisher, Cat# 140675). Cells were exposed to gap junction permeable Lucifer yellow (1.5 mg/mL) (457 Da) (MilliporeSigma, Cat# L0259-25MG) and the gap junction impermeable dextran rhodamine B (0.5 mg/mL) (10,000 Da) (ThermoFisher, Cat# D1824) in PBS. Each well was scraped in three distinct locations using a scalpel blade and incubated for three minutes at 37 °C and 5% CO_2_. Subsequently, the PBS-dye solution was aspirated, and cells were washed three times with PBS and fixed with 10% neutral-buffered formalin for 15 min at room temperature. Three random images were captured along each scrape line using a Zeiss LSM 800 confocal Airyscan microscope equipped with a 10× objective. The distance of Lucifer yellow transfer was measured from the scrape edge using ImageJ, and data were analyzed using a one-way ANOVA with Dunnett’s multiple comparisons test.

### 2.5. Dual Whole Cell Patch Clamp

On the day of patch clamp, REKs and Cx43-ablated REKs were trypsinized, plated onto glass coverslips, and incubated for 2 h at 37 °C and 5% CO_2_. Coverslips were transferred to a recording chamber, washed, and bathed in extracellular solution containing 140 mM NaCl, 2 mM CsCl, 2 mM CaCl_2_, 1 mM MgCl_2_ 5 mM HEPES, 4 mM KCl, 5 mM D-Glucose, and 2 mM pyruvate at pH 7.4. Glass micropipettes were pulled with a PC-100 puller (Narishige, Amityville, NY, USA) and filled with intracellular solution composed of 130 mM CsCl, 10 mM EGTA, 0.5 mM CaCl_2_, 3 mM MgATP, 2 mM Na_2_ATP, and 10 mM HEPES at pH 7.2 [42]. The patch pipette resistance was typically 2–3 MΩ. Isolated cell pairs were selected for dual whole cell patch clamp, with each of the cells under voltage clamp at 0 mV. One cell in the pair received seven second duration transjunctional voltage (*V_j_*) pulses ranging from ±20 to ±100 mV (at 20 mV increments), while the other cell of the pair remained at a constant holding potential of 0 mV to record junctional currents (*I_j_*), as previously described [42]. Macroscopic junctional conductances (*G_j_*) were calculated from the *I_j_* recorded during a ±20 mV *V_j_* pulse (*G_j_* = *I_j_ / V_j_*) [43,44].

### 2.6. Propidium Iodide Dye Uptake Assay

Hemichannel function was assessed by a dye uptake assay, similar to Tong et al. (2007) [45]. Connexin-deficient HeLa cells were seeded on glass coverslips in six-well plates at low density, transfected, and incubated for 24 h at 37 °C and 5% CO_2_. Growth media was aspirated, and cells were gently washed twice with divalent cation-free extracellular solution (DCF-ECS) (140 mM NaCl, 5.4 mM KCl, 2 mM EGTA, 10 mM HEPES, 25 mM D-glucose, osmolarity 298 mOsm, pH 7.35) and incubated in 1 mL DCF-ECS containing 0.15 mM propidium iodide (PI; 668.4 Da) (ThermoFisher, Cat#P1304MP) for 15 min. The incubation solution was then aspirated, and the cells were gently washed three times with regular extracellular solution (ECS) (140 mM NaCl, 5.4 mM KCl, 1.4 mM MgCl_2_, 2 mM CaCl_2_, 10 mM HEPES, 25 mM D-glucose, osmolarity 298 mOsm, pH 7.35). Cells were subsequently fixed with 4% paraformaldehyde for 20 min at room temperature, nuclei counterstained with Hoechst 33342, and the coverslips mounted onto glass microscope slides using Airvol mounting medium. Images were captured on a Zeiss LSM 800 confocal Airyscan microscope equipped with a 10× objective lens. A third-party investigator, blinded to the treatment, was used to quantitate the percentage of GFP-positive connexin-expressing cells loaded with PI. Between 30 and 160 cells were counted for each coverslip in each experimental group.

### 2.7. Brefeldin A Treatment

To assess the clearing of wildtype and variant gap junction plaques, 24 h after transfection, Cx43-ablated REKs were treated with 2 μg/mL brefeldin A (BFA) (MilliporeSigma, Cat# B5936) for up to 7 h at 37 °C and 5% CO_2_ to inhibit anterograde protein transport between the ER and Golgi apparatus. Coverslips were collected at 1- or 2-h intervals and cells were fixed in ice cold 80% methanol/20% acetone at 4 °C for 10 min and nuclei stained with Hoechst 33342. Images were captured using a Zeiss LSM800 Airyscan confocal microscope equipped with a 63× oil immersion objective lens. Between 10 and 90 GFP-positive cell pairs were assessed at each timepoint, with a total of five biological replicates performed. A third-party investigator that was blinded to the treatment quantified the number of Cx43 gap junction plaques between GFP-positive cell–cell pairs.

### 2.8. Cycloheximide Treatment and Western Blot

Cx43-ablated REKs expressing wildtype or variant Cx43 were treated with 10 μg/mL cycloheximide (CHX; MilliporeSigma, Cat# C7698) for 0, 1, 3, 5, or 7 h at 37 °C and 5% CO_2_ to inhibit protein synthesis. At each time point, cell lysates were prepared on ice using RIPA lysis buffer (150 mM NaCl, 1% Triton X-100, 0.5% sodium deoxycholate, 0.1% sodium dodecyl sulphate, 50 mM Tris, pH 8.0) supplemented with 100 mM NaF, 100 mM NaVO_4_, and a complete™ mini protease inhibitor cocktail tablet (MilliporeSigma, Cat# 11836153001). Protein concentrations were subsequently determined using a bicinchoninic acid assay kit (ThermoFisher, Cat# 23225). Proteins were transferred from a 10% SDS-PAGE gel onto nitrocellulose membranes using the iBlot^®^ dry-transfer system (ThermoFisher). Membranes were blocked in 5% skim milk and incubated with rabbit polyclonal anti-Cx43 antibody (MilliporeSigma, Cat# C6219) (1:5000) and mouse anti-glyceraldehyde-3-phosphate dehydrogenase antibody (GAPDH; MilliporeSigma, Cat# MAB374) (1:5000) at 4 °C overnight. The following day, membranes were washed and labeled with goat anti-rabbit IgG IRDye 800 (Rockland Immunochemicals, Pottstown, PA, USA, Cat# 611-132-002) (1:5000) and goat anti-mouse IgG Alexa Fluor 680 (Life Technologies, Burlington, ON, Canada, Cat# A21057) (1:5000) for 45 min at room temperature. Protein bands were visualized and quantified using the Odyssey LiCor infrared imaging system. A total of three to six replicates were performed. The ratio of Cx43/GAPDH was calculated for each time point and normalized to 0 h for each treatment. Data were analyzed using a one-way ANOVA with a Dunnett’s post-hoc test.

## 3. Results

### 3.1. Cx43 Is the Major Contributor to GJIC in REKs

**Figure 1:**
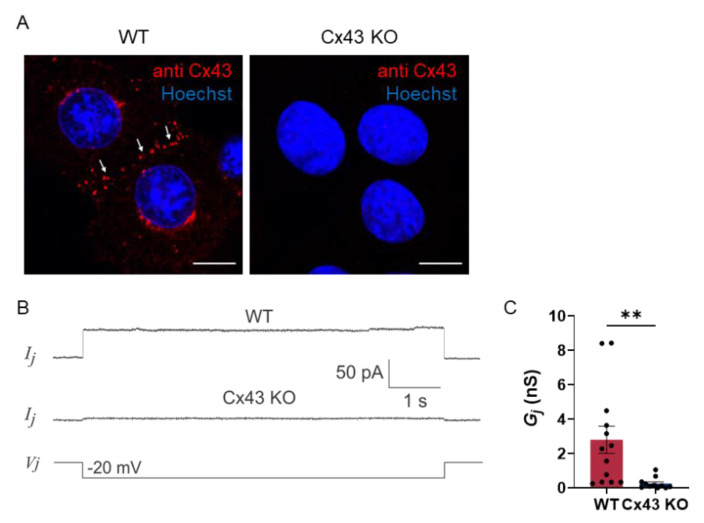
Ablation of Cx43 significantly reduces electrical coupling in REKs. (**A**) Prototypical Cx43-positive gap junctions (arrows) were readily detected in wildtype REKs (WT) but not evident in Cx43-ablated (Cx43 KO) REKs. Nuclei were labeled with Hoechst 33342 (blue). Scale bars = 10 μm. (**B**) Representative junctional current (*I_j_*) recordings in response to a −20mV voltage pulse in WT or Cx43–KO REK pairs. (**C**) Quantification of the macroscopic junctional conductance (*G_j_*) reveals that ablation of Cx43 significantly reduces electrical coupling in keratinocytes. ** *p* < 0.01.

We have previously shown that the CRISPR/Cas9 ablation of Cx43 significantly reduces the ability of REKs to pass dye [40]. Here, we show that Cx43 was undetected in Cx43 knockout (KO) REKs but was readily observed in gap junctions between wildtype REKs (Figure 1A, arrows). Dual whole cell patch clamp was used to record *I_j_* for calculation of junctional coupling conductance (*G_j_*). Cx43–KO REK cell pairs showed significantly reduced *G*_j_ compared to those of wildtype REKs (*p* < 0.01) (Figure 1B,C). Notably, Cx43-KO REKs retained low levels of *G_j_*s, including some *I_j_*s, showing voltage-dependent gating characteristic of gap junctions (data not shown), likely contributed by infrequent expression of other keratinocyte connexins. These findings reveal that Cx43 is the major contributor to the overall level of GJIC in REKs.

### 3.2. Cx43 Variants Traffic and Assemble into Gap Junctions in REKs Independent of Endogenous Cx43

**Figure 2:**
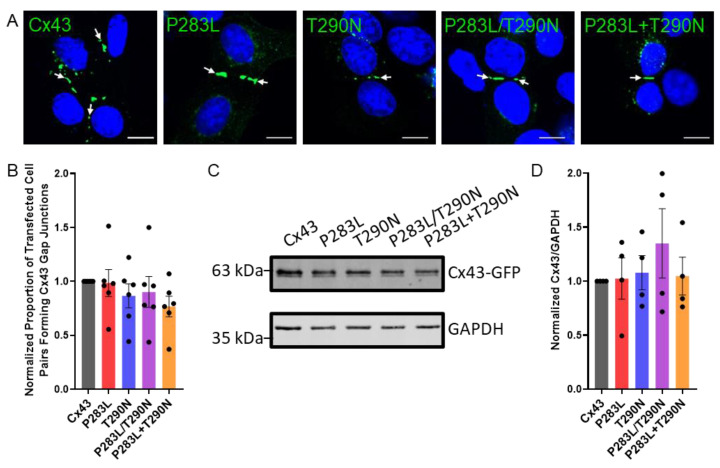
Cx43 variants traffic and assemble effectively into prototypical gap junction plaques in Cx43–KO REKs. (**A**) Cx43 KO REKs engineered to express GFP-tagged EKVP-linked Cx43 variants (green) formed prototypical gap junctions at cellular interfaces (arrows). Nuclei were demarcated with Hoechst 33342 (blue). (**B**) Quantification revealed that cells expressing EKVP-linked Cx43 variants were equally effective at forming gap junctions as wildtype Cx43. (**C**,**D**) Western blotting for Cx43 revealed that the variants were equally expressed compared to wildtype Cx43 in Cx43–KO REKs. Immunoblotting for GAPDH was performed as a gel loading control. Scale bars = 10 μm.

Previously, we demonstrated in a number of reports that GFP-tagging of Cx43 had no significant detrimental effect on the trafficking and assembly of Cx43 into gap junctions [40,46,47], thus we performed all experiments using GFP-tagged Cx43 and variants. High resolution fluorescence imaging revealed that Cx43-ablated REKs expressing either wildtype or EKVP-associated Cx43 variants in isolation (P283L and T290N alone) or in combination (i.e., P283L/T290N mutations within the same plasmid or P283L + T290N mutations engineered on separate plasmids) formed prototypical gap junctions (Figure 2A, arrows). Quantification of the proportion of transfected cell pairs that formed Cx43 gap junction plaques at cell–cell interfaces revealed that wildtype and Cx43 variants were equally efficient at forming gap junctions (Figure 2B). It is also notable that the variants did not change the integrity of the cells, as there was no evidence of dead or dying cells as revealed by the absence of cell blebbing or pyknotic nuclei. Western blotting for Cx43 revealed that Cx43-KO REKs efficiently, and approximately equally, expressed GFP-tagged Cx43 and variants (Figure 2C,D). Since the EKVP-associated variants being examined here are inherited in an autosomal dominant manner, we wanted to assess whether the presence of endogenous Cx43 would affect their ability to form gap junctions. To that end, all variants assessed in isolation or combination readily assembled into gap junctions when expressed in wildtype REKs that are rich in endogenous Cx43 (Figure S1).

**Figure 3:**
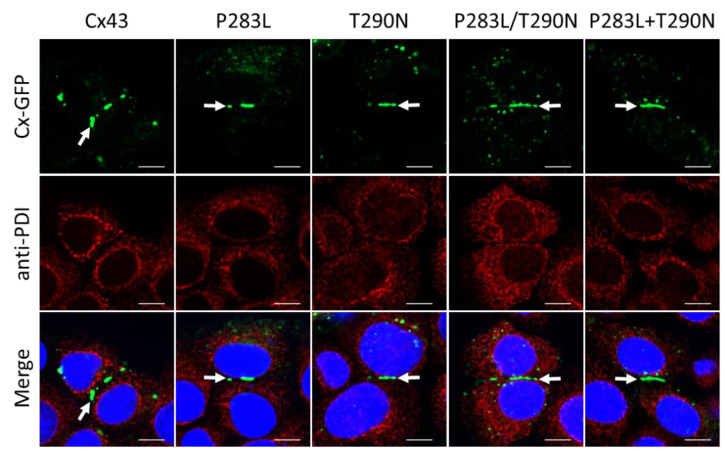
Cx43 variants were not detected in the endoplasmic reticulum of Cx43–KO REKs. Wildtype Cx43 or Cx43 variants (green) do not co-localize with protein disulfide isomerase (PDI; red), a resident endoplasmic reticulum protein. Arrows indicate Cx43 gap junctions at cell-cell interfaces. Nuclei were labeled with Hoechst 33342 (blue). Scale bars = 10 µm.

**Figure 4:**
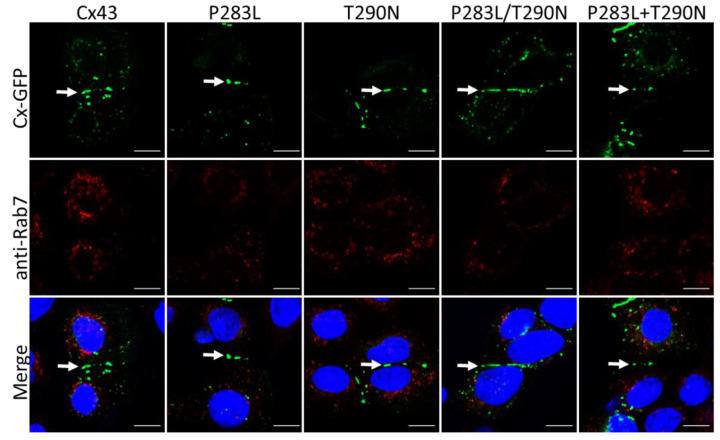
Intracellular Cx43-positive structures do not localize with Rab7-positive endosomes. Wildtype or Cx43 variants (green) do not co-localize with Rab7 (red), a resident endosomal protein. Nuclei are labeled with Hoechst 33342 (blue). Arrows denote gap junctions. Scale bars = 10 μm.

The initial case report using patient biopsies suggested the Cx43 variants were possibly intracellularly retained [18]. While this was not a prevailing feature in our study, Cx43 or variant expressing REKs were counterstained for protein disulfide isomerase (PDI) (Figure 3), GM130 (Figure S2), or Rab7 (Figure 4), respectively. High-resolution fluorescence microscopy revealed that wildtype or Cx43 variants were rarely identified in the ER, Golgi apparatus, or Rab7-positive endosomes. Collectively, these findings suggest that EKVP-associated variants are not trafficking-defective and are readily transported to the cell surface for gap junction assembly when expressed in REKs.

### 3.3. Wildtype Cx43 and Variants Enhance Dye Transfer in Cx43-KO REKs

**Figure 5:**
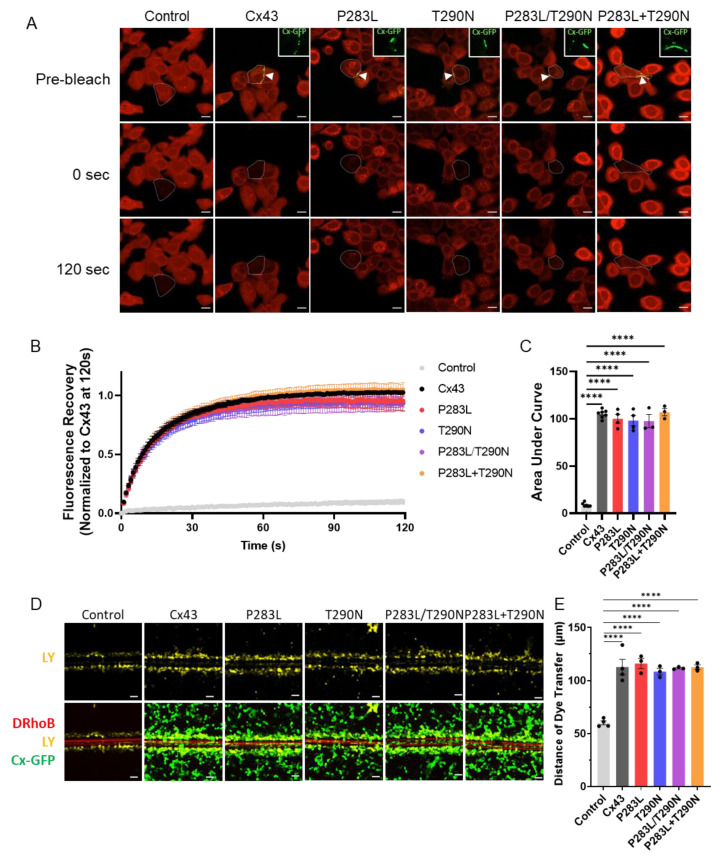
Re-expression of wildtype or Cx43 variants enhance dye transfer in Cx43-KO REKs. (**A**) Cx43 KO REKs engineered to express GFP-tagged wildtype or Cx43 variants (green, insets taken from site of arrowheads) were loaded with calcein red-orange AM (red). Single cells (outlined) in small clusters with optically detectable gap junctions (arrowheads) were photobleached. Representative images of cells prior to photobleaching, immediately following photobleaching (0 s), and after a 120 s recovery period. Scale bars = 10 μm. (**B**) Fluorescence recovery mediated by gap junctional transfer of dye from adjacent cells was monitored over 120 s and normalized to Cx43 at 120 s. (**C**) Quantification of the area under the curve revealed that re-expression of wildtype or Cx43 variants enhanced dye transfer in Cx43-ablated REKs. (**D**) Representative images of Cx43–KO REKs expressing GFP-tagged wildtype or Cx43 variants (green) were scraped-loaded with Lucifer yellow (LY, yellow) and dextran rhodamine B (DRhoB, red). Scale bars = 50 μm. (**E**) The distance of LY dye spread from the scrape edge was significantly increased in keratinocytes expressing wildtype or Cx43 variants compared to control Cx43-KO REKs. **** *p* < 0.0001.

We next used fluorescence recovery after photobleaching (FRAP) to assess the functional status of gap junctions formed by Cx43 variants. Briefly, single cells in small clusters of Cx43 or variant expressing cells loaded with a gap junctional permeable dye were bleached and fluorescence recovery was tracked and quantified. Representative images showcase calcein red-orange fluorescence in Cx43–KO REKs pre-bleaching, immediately after photobleaching, and after a two-minute recovery period (Figure 5A). Fluorescence recovery was plotted as a function of time normalized to the level of fluorescence recovery of wildtype Cx43 at 120 s (Figure 5B). Quantification of the area under the curve revealed that re-expression of wildtype Cx43 and all Cx43 variants significantly restored dye transfer in Cx43-KO REKs (*p* < 0.0001) (Figure 5C). Calcein red-orange, while easily optically distinguished from the GFP fluorescent tag of Cx43 and variants, is a relatively large dye (570 Da), which may restrict its passage through Cx43 channels. Thus, we also tested the ability of Cx43 variants to pass the smaller molecular weight dye, Lucifer yellow (457 Da), in a scrape load assay where dextran rhodamine B (10,000 Da) was used to demarcate damaged cells at the scrape edge (Figure 5D). Cx43-KO REKs expressing wildtype Cx43 or Cx43 variants exhibited a significantly greater degree of Lucifer yellow dye spread than control Cx43-KO REKs (*p* < 0.0001) (Figure 5E). Taken together, these results suggest that EKVP-associated variants form functional gap junction channels.

### 3.4. Cx43 Variants Do Not Form Hyperactive Hemichannels

**Figure 6:**
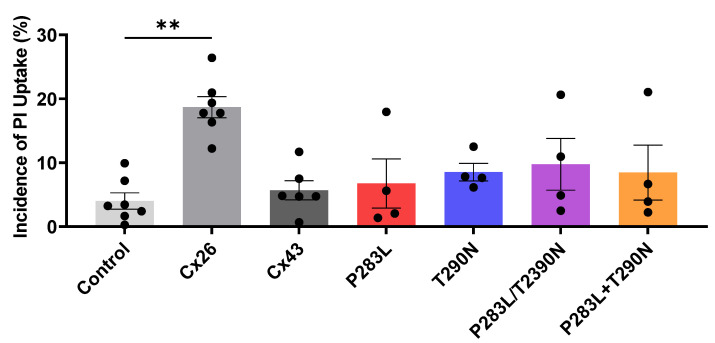
Cx43 variants do not form hyperactive hemichannels permeable to propidium iodide (PI). HeLa cells engineered to express wildtype or Cx43 variants were incubated in membrane-impermeable PI in extracellular media lacking regulatory divalent cations designed to open hemichannels. HeLa cells expressing Cx26 were used as positive control for hemichannel-mediated dye uptake. Quantification of the incidence of dye uptake indicated that neither wildtype Cx43 nor Cx43 variants hemichannels were opened to a pathological state where they were capable of passing PI. ** *p* < 0.01.

Since augmented hemichannel function has been reported for other EKVP-linked mutants [23], we wanted to assess if the removal of extracellular divalent cations would lead to a potential pathological level of variant Cx43 channel opening in culture cells. HeLa cells were engineered to express wildtype or Cx43 variants, and hemichannel function was determined by assessing propidium iodide (PI) dye uptake in the absence of regulatory divalent cations. Since wildtype Cx43 hemichannels are not highly responsive to divalent cation-free environments, we used Cx26 as a positive control to demonstrate that the divalent cation free extracellular medium was indeed able to open a connexin hemichannel (Figure 6). Quantification of the incidence of PI uptake revealed that, while HeLa cells expressing Cx26 demonstrated significant dye uptake, both wildtype and Cx43 variants did not increase hemichannel-mediated dye uptake compared to control HeLa cells (Figure 6). These studies suggest that any Cx43 variant hemichannels that had formed in HeLa cells were not in a constituently open state that could be detected by dye uptake.

### 3.5. The P283L Variant Persists Longer in Gap Junctions than Wildtype Cx43

**Figure 7:**
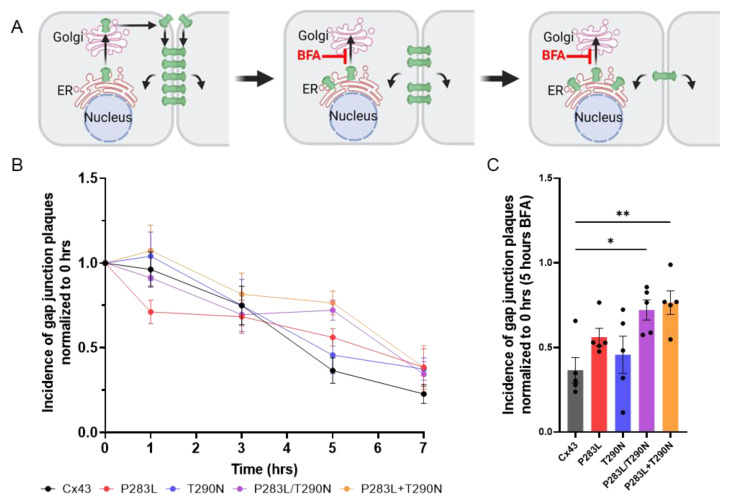
The P283L and T290N variants in combination prolong the clearing of Cx43 gap junctions. (**A**) Schematic illustrating the site of brefeldin A (BFA) action in blocking protein secretion. (**B**) Quantification of gap junction plaques observed in Cx43–KO REKs expressing GFP-tagged wildtype or Cx43 variants after treating with BFA from 0 to 7 h. (**C**) Incidence of gap junction plaques found at cell–cell interfaces 5 h after BFA treatment * *p* < 0.05; ** *p* <0.01. Schematic in panel **A** was created using BioRender.com.

Since the EKVP-associated variants occur in a motif of Cx43 that is the binding site for several members of the interactome and a domain known to regulate Cx43 internalization and degradation (Table 1) [31,32,33,34,35,36,37,38], we next sought to assess the resident time of wildtype and Cx43 variants within gap junctions at the cell surface. When brefeldin A (BFA) was used to reversibly block protein secretion at the level of the ER/Golgi apparatus, the clearing of gap junctions composed of wildtype and Cx43 variants was tracked over a seven-hour treatment (Figure 7A,B). Gap junctions composed of the P283L and T290N variants had statistically longer residency time at the cell surface after 5 h of BFA treatment compared to wildtype Cx43, while gap junctions composed of P283L appeared to have a longer residency, but this was just below statistical significance. This finding suggests that a combination of the two variants is affecting Cx43 clearing from the cell surface and may have implication in the EKVP phenotype.

**Figure 8:**
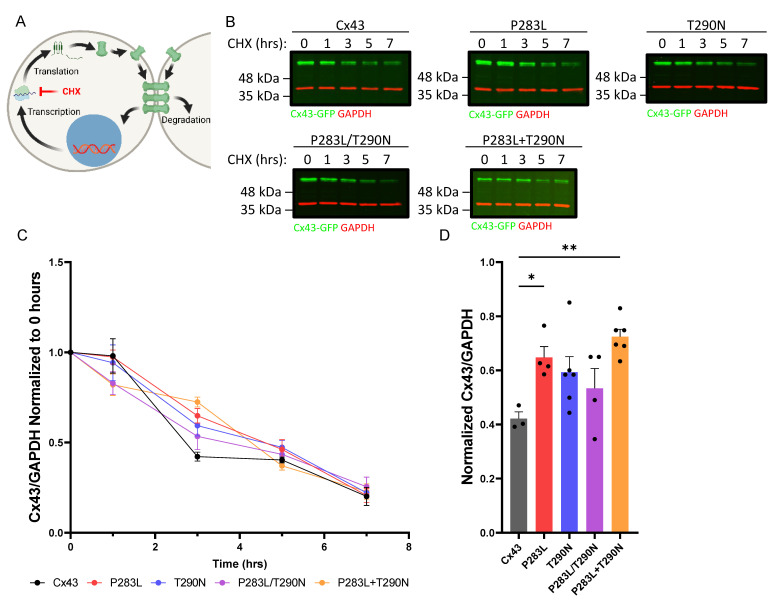
The P283L variant contributes to a slower degradation of Cx43. (**A**) Illustration of cycloheximide (CHX) inhibition of protein synthesis in the context of the Cx43 lifecycle from transcription through to internalization and degradation. (**B**) Immunoblots of Cx43–KO REKs expressing GFP-tagged wildtype or Cx43 variants (green) treated with CHX for 0 to 7 h. GAPDH (red) was used as an internal control. (**C**) The Cx43/GAPDH ratio was calculated and normalized to 1. (**D**) After 3 h of CHX treatment, the P283L and P283L + T290N variants were found to be significantly higher than wildtype Cx43. * *p* < 0.05; ** *p* < 0.01. Schematic in panel **A** was created using BioRender.com.

The question remained as to whether the P283L and/or T290N variants would exhibit a delay in Cx43 degradation compared to wildtype Cx43. To address the relative degradation of Cx43 we blocked protein synthesis with cycloheximide (CHX) for up to seven hours and tracked the loss of wildtype and Cx43 variants by immunoblotting for Cx43 (Figure 8A,B). In keeping with Cx43 having a half-life of 2–3 h in cultured cells [48], we found that wildtype Cx43 was readily degraded in cultured REKs validating this approach (Figure 8C). Interestingly, the P283L variant and a combination of the P283L + T290N variants (co-transfected with two separate variant-encoding plasmids) showed significantly delayed degradation after three hours of CHX treatment, further pointing to the notion that the P283L variant was impacting the normal clearing and degradation of Cx43.

## 4. Discussion

Keratinocytes differentially express at least eight connexin isoforms that act to create a dynamic gap junctional network within the epidermis that regulates epidermal homeostasis. Cx43 is the most abundant connexin in the epidermis, with high levels of expression in the basal and spinous layers that taper off in the granular layer [3,5,6,14]. It is apparent that Cx43 plays a pivotal role in the epidermis, as highlighted by a fatal defect in barrier function in genetically-modified mice harboring a C-terminal truncated mutant of Cx43 [49] and the association between some Cx43 mutations and skin disorders [17,18,20,21,22,50,51,52,53,54,55,56]. We previously showed that ablation of Cx43 from REKs significantly impairs keratinocyte differentiation into organotypic epidermis, concomitant with a reduction in gap junction-mediated dye transfer between keratinocytes [40]. In the present study, we found that electrical coupling between pairs of Cx43-ablated keratinocytes is significantly reduced, but not abolished, establishing that Cx43 is the major contributor to the overall level of GJIC in cultured keratinocytes. Since REKs mimic basal keratinocytes in vivo by being proliferation, migration and differentiation-competent [40,41], we propose that Cx43 is likely accounting for the majority of GJIC in rat basal keratinocytes in situ.

Currently, four autosomal dominant mutations/variants in the *GJA1* gene (encoding Cx43) have been clinically associated with EKVP [17,18]. The A44V and E227D mutations have been shown to exhibit augmented hemichannel function, which is likely informing on the mechanistic root of EKVP in patients harbouring these mutants [23]. The more recently discovered de novo *GJA1* variants (encoding P283L and T290N) have been reported in only two patients [18], so it remains unclear if one or both of these variants are indeed causal of EKVP. It is notable that the one patient that harbors both the P283L and T290N variants has more severe EKVP than the patient that expresses only the P283L variant [18] raising questions as to whether there is any additive or synergistic relationship between the genotype and phenotype.

Localization of Cx43 in skin biopsies from EKVP patients raised the possibility that one or both of these variants were retained within an intracellular compartment [18]. High resolution imaging of the P283L and T290N variants expressed in keratinocytes revealed that they assembled into prototypical gap junctions in the presence or absence of endogenous Cx43. Thus, not unlike the clinical report describing the A44V and E227D mutants as potentially trafficking defective [17], being later shown to assemble into gap junctions when overexpressed in HeLa cells [23], we conclude that both P283L and T290N variants can readily traffic from the ER though the Golgi apparatus to form gap junctions at the cell surface in tissue-relevant REKs. Our initial studies using a dual whole cell patch clamp to determine if the variants formed functional gap junctions in REKs resulted in cell pairs that had junctional conductances that exceeded 50 nS, masking the distinction between excellent gap junction coupling and cytoplasmic bridging that may exist between REKs that were undergoing cell division (data not shown). However, FRAP and scrape loading dye transfer studies engaging two distinctly different dyes (calcein red-orange and Lucifer yellow) consistently revealed that both variants exhibited GJIC levels that were comparable with wildtype Cx43. The fact that the P283L and T290N variants formed functional gap junctions aligns well with the previous finding that the A44V and E227D EKVP-associated mutants formed functional gap junctions with similar unitary channel conductance and voltage gating properties as reported for wildtype Cx43 [23]. However, A44V and E227D hemichannels adopted open configurations more frequently and for a longer duration than wildtype Cx43 hemichannels [23]. In our studies, the P283L or T290N variants did not acquire a detectable constitutively open or leaky hemichannel state. This is consistent with variant expressing cells lacking any evidence of necrosis or apoptosis in the event that leaky hemichannels were releasing vital members of the metabolome necessary for cell survival. We cannot rule out that more subtle changes are occurring at the hemichannel level, but this awaits an electrophysiological assessment in a defined culture system where channels formed from other integral membrane proteins, such as endogenous hemichannels, can systematically be eliminated.

Cx43 undergoes continuous and rapid turnover with a half-life of 1–3 h [48,57,58,59], and changes in connexin half-life kinetics may have linkages to the onset of developmental abnormalities and disease [38,60,61,62]. It is also known that the C-terminal domain of Cx43 has a large interactome that includes binding sequences for NEDD4, an E3-ubiquitin ligase, and AP-2, that are likely all involved with Cx43 internalization [31]. Interestingly, the P283L and T290N variants reside in or near the PPxY motif important in regulating Cx43 sorting and internalization [31,36]. In the present study, we showed that keratinocytes co-expressing both Cx43 variants displayed significantly delayed kinetics of gap junction removal, suggesting that they reside in a motif that is important for gap junction clearing from the cell surface. It is likely that the P283L variant plays a larger role in delaying Cx43 gap junction internalization, as it was previously found to have modestly higher steady-state levels than wildtype Cx43 when transiently expressed in SKHep1 cells [36]. Since internalization of variant-containing gap junctions was delayed, we predicted that one or both variants might lead to a delay in degradation in the presence of CHX. This turned out to be the case for the P283L variant, but not the T290N variant. This finding is generally in keeping with a recent study in culture cells and zebrafish where a ∆256–289 mutant caused dysregulated Cx43 gap junction endocytosis resulting in a longer Cx43 protein half-life, elevated levels of Cx43, more numerous and larger gap junction plaques, and increased GJIC that coincided with severe cardiovascular defects [38]. Thus, we deduced that the P283L variant has a significant impact on the fate of Cx43. The T290N variant appears to further augment Cx43 internalization and its residency time on the cell surface when found in combination with the P283L variant.

We can only speculate on how prolonged Cx43 residency within functional gap junctions might disrupt the homeostasis of the epidermis. The fact that delayed Cx43 removal leads to cardiovascular defects in zebrafish suggests that potentially too much GJIC can lead to a pathology [38]. In the skin, dysregulated control of Cx43 turnover may change the delicate balance between keratinocyte proliferation and differentiation. For example, extended Cx43-based cell coupling may allow basal keratinocytes to remain in a prolonged proliferative state, delaying keratinocyte differentiation, thereby giving rise to the hyperkeratosis seen in EKVP. That said, it remains unclear how these Cx43 variants might be linked to erythema. The short half-life of Cx43 is thought to allow cells to quickly alter the extent of GJIC, thus any delay in this process could lead to a dysregulated response to pathogenic stimuli, leading to inflammation and erythema.

Caution needs to be exercised in concluding that the observed modest delay in Cx43 gap junction clearing and/or degradation found in our study causes EKVP observed in patients harboring the P283L or P283L + T290N variants. Other mechanisms that need to be considered include the possibility that Cx43 variants gain other functions, such as the capacity to interact with any of the other half-dozen or more connexin isoforms found in the epidermis [3]. However, even if that was the case, since the variants are functional it is not clear how that would lead to a pathology. Since whole exome sequencing does not appear to have been performed on these EKVP patients, other genetic causes of disease need to be considered and ruled out, as a number of genes unrelated to connexins have been linked to the heterogeneity of EKVP diseases [26,27,28,29,30]. Nevertheless, the variant-linked prolonged persistence of Cx43 gap junctions and modest delay in degradation highlights yet another possible mechanism underpinning EKVP.

EKVP pathogenesis is complex, involving at least three connexin isoforms (Cx30.3, Cx31, and Cx43) that are temporally and spatially expressed in the epidermis [3]. Similar to congenital cataracts linked to Cx50 and Cx46 [63], EKVP represents another disease linked to multiple connexins, raising questions as to what mechanistic feature of each connexin variant leads to the same clinical disease. Already, two mechanisms have been proposed that include augmented hemichannel function [23,24], defective trafficking [64,65], and now, possible alterations in gap junction fate and degradation. As there is a lack of effective treatments for EKVP, it is critical to characterize all known connexin variants associated with EKVP to help shape the development and deployment of strategically targeted treatments for this disorder.

## Figures and Tables

**Table 1:** Cx43 protein binding motifs near the site of the EKVP associated variants. The proline residue at position 283 is underlined.

Cx43 Motifs	Binding Protein	References
^263^QKYAYFNGCSSPTAPLSPMS^282^	Hsc70	[31,37]
^266^AYFNGCSSPTAPLSPMSP^283^	c-Src/PTEN/Csk	[31,33,34,35]
^283^PPGY^286^	NEDD4	[31,32]
^286^YKLV^289^	µ2 subunit of AP-2	[31,36]

## Supplementary Materials

The following supporting information can be downloaded at: https://www.mdpi.com/article/10.3390/ijms23010486/s1.
