# Supplementary material for: Interrogation of Carboxy-Terminus Localized GJA1 Variants Associated with Erythrokeratodermia Variabilis et Progressiva
Source: Int J Mol Sci. 2022 Jan 1;23(1):486. doi: 10.3390/ijms23010486 (PMC8745721; doi:10.3390/ijms23010486)
Supplement: Supplementary file 1 [file ijms-23-00486-s001.zip › ijms-1413388-supplementary-done.pdf]

Supplementary Materials

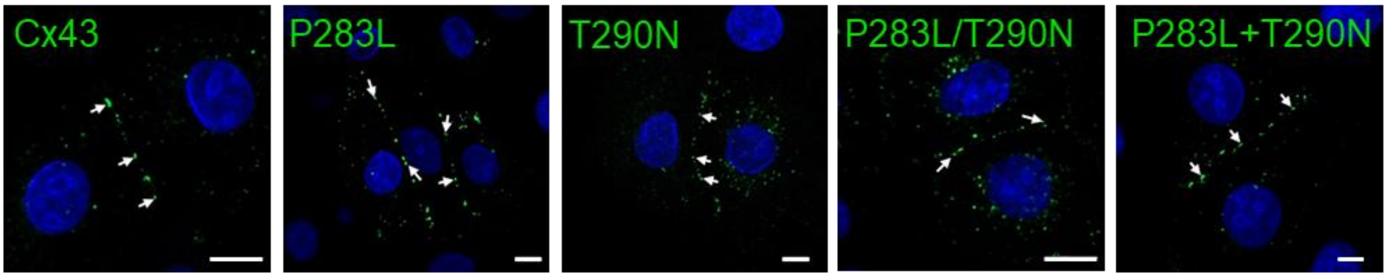

Figure S1. Cx43 variants traffic and assemble into prototypical gap junctions in REKs that endogenously express Cx43. (A) REKs engineered to express GFP-tagged EKVP-linked Cx43 variants (green) formed prototypical gap junctions at cellular interfaces (white arrows). Nuclei were counterstained with Hoechst 33342 (blue). Scale bars = 10 μm.

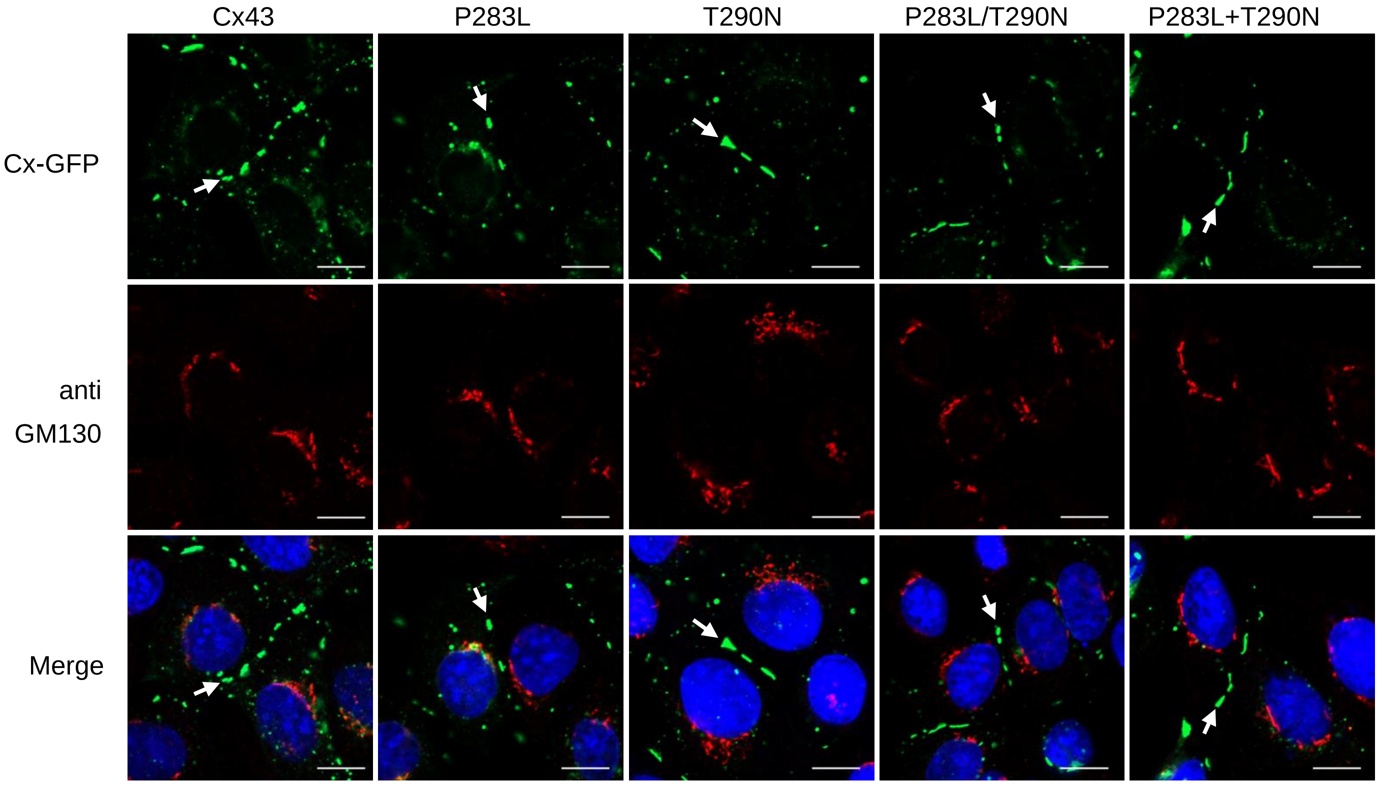

Figure S2. Cx43 variants do not notably localize to the Golgi apparatus. Wildtype or Cx43 variants (green) do not co-localize with GM130 (red), a resident Golgi apparatus protein. Arrows indicate gap junctions formed by wildtype or Cx43 variants. Nuclei are labeled with Hoechst 33342 (blue). Scale bars = 10 μm.
